# An *Enterobacter cloacae* strain NG-33 that can solubilize phosphate and promote maize growth

**DOI:** 10.3389/fmicb.2022.1047313

**Published:** 2022-11-10

**Authors:** Xinxin Chen, Caihe Yang, Jairo A. Palta, Youzhi Li, Xianwei Fan

**Affiliations:** ^1^State Key Laboratory for Conservation and Utilization of Subtropical Agro-bioresources, College of Life Science and Technology, Guangxi University, Guangxi, Nanning, China; ^2^UWA School of Agriculture and Environment, The UWA Institute of Agriculture, The University of Western Australia, Perth, WA, Australia; ^3^CSIRO Agriculture and Food, Wembley, WA, Australia

**Keywords:** *Enterobacter cloacae* strain NG-33, phosphate-solubilizing bacteria, maize, promoting growth, organic acids

## Abstract

It is critical to identify and evaluate efficient phosphate-solubilizing bacteria (PSB) that enable P uptake from unavailable forms, and therefore improve the phosphorus (P) uptake efficiency of crops. The *Enterobacter cloacae* strain NG-33, belonging to PSB, was isolated and identified from calcareous rhizosphere soils in Nonggang National Reserve, Guangxi, China. The stain NG-33 could reduce the pH of the medium to below 5.6, and had the ability to release soluble phosphorus (P; 180.7 μg ml^−1^) during the culture in the National Botanical Research Institute’s Phosphate medium (NBRIP), and produced such organic acids as gluconic acid (4,881 mg L^−1^), acetic acid (346 mg L^−1^), and indole-3-acetic acid (20.4 μg ml^−1^). It could also convert inorganic P in AlPO_4_ (Al-P) and FePO_4_ (Fe-P) into soluble P, with conversion efficiencies of 19.2 μg ml^−1^ and 16.3 μg ml^−1^, respectively. Under pot experiments and when compared controls without inoculating NG-33, the shoot and root biomass of maize seedlings showed increases by 140% for shoot biomass and by 97% for root biomass in loamy soil (P sufficient) inoculated with NG-33. In sandy soil (P deficit) supplemented with tricalcium phosphate and inoculated with NG-33, the soluble P content was significantly higher, 58.6% in soil and 33.6% in roots, meanwhile, the biomass of shoots and roots increased by 14.9 and 24.9%, respectively. The growth-promoting effects coupled to the significant increase in leaf net photosynthetic rate and stomatal conductance of plants grown in NG-33-inoculated soil. Inoculating NG-33 could significantly improve the diversity and richness of bacterial population and altered the dominant bacterial population in soil.

## Introduction

Phosphorus (P) deficiency is a major limiting factor for crop growth and yield in most agricultural soils (Schachtman et al., 1998). However, most of P in agricultural systems are unavailable for plants because they are usually present in mineral P and/or in organic compounds. In general, plants require at least 30 μmol l^−1^ of P to maintain maximum productivity (Daniels et al., 2009), and the concentration of soluble P (1 ppm or less) and the rate of diffusion is very low (10^−12^ to 10^−15^ m^2^ s^−1^) in many soils (Goldstein, 1994; Schachtman et al., 1998). Therefore, phosphate fertilizer (from phosphate rock) is needed to increase growth and yield of crops.

Some microbes can not only enhance the release of immobile P from tricalcium phosphate, dicalcium phosphate, hydroxyapatite, and rock phosphate (Kim et al., 1997; Perez et al., 2007; Hameeda et al., 2008) but also immobilized P (Schachtman et al., 1998; Otieno et al., 2015), depending on species and strains. Many bacterial species have been reported to promote crop growth by increasing P availability, such as *Pseudomonas* sp. EAV, *Arthrobacter nicotinovorans* EAPAA (Pereira and Castro, 2014), *P*. *fluorescens* L321 (Otieno et al., 2015), *Serratia marcescens* EB 67 (Hameeda et al., 2008), *P*. *putida* CO1 and *Bacillus paramycoides* CO8 (Pandey and Gupta, 2020), *B.* sp. B17 and *Burkholderia* sp. B5 (Oliveira et al., 2009), and *Azotobacter*, *Rhizobium*, *Azospirillum*, and *Alcaligenes* (Rodríguez and Fraga, 1999; Trivedi and Sa, 2008). However, few have been isolated, and little is known about roles of *Enterobacter* strains with promoting plant growth by increasing phosphate solubilization (Shahid et al., 2012).

As for microbes, their mechanisms on P solubilization are involved in such enzymes as phosphatases and phytases (Yadav and Tarafdar, 2003; Aseri et al., 2009; Maougal et al., 2014) and various organic acids (e.g., gluconic, acetic, fumaric, lactic/succinic, citric, oxalic, and propionic; Rashid et al., 2004; Marciano Marra et al., 2012; Shahid et al., 2012). The biodiversity and composition of soil organisms (phosphate-solubilizing bacteria, nitrogen-fixing bacteria, etc.) are tightly associated with the soil healthy (Anggrainy et al., 2020), and can also induce changes in plant productivity (Wagg et al., 2014). The bacterial community structure are determinative with the changes of soil pH and depth in subarctic tundra soil (Kim et al., 2014). However, the changes in the bacterial community and composition in inoculated soil remain considerably unclear (Zhao et al., 2022).

In this study, we report the P solubilization and growth promoting effects on maize (*Zea mays* L) of *E. cloacae* strain NG-33 and the mechanisms.

## Materials and methods

### Isolation of phosphate-solubilizing bacteria

Rhizosphere soil was sampled from plants (*Ophiopogon platyphyllus* Merr. et Chun) grown in the Nonggang National Reserve (Guangxi, China; 106°42′28′′E, 22°13′56′′N, 500 ft. elevation) according to methods in the literature (Sun et al., 2020), where the soil is weakly alkaline (pH 7.2–7.8). In brief, sampled soil was ground in a mortar, and then suspended in sterile water. Gradient-diluted soil suspension was plated on the medium containing the National Botanical Research Institute’s Phosphate (NBRIP) and then cultured at 37°C for 72 h (Nautiyal, 1999). The isolates of bacteria from culture were subjected to pure cultivation on minimal medium following the methods described by Katznelson et al. (1962). The phosphorus-solubilizing isolates were identified through cultivation in the liquid NBRIP media with iron phosphate (Fe-P) and aluminum phosphate (Al-P) as the sole metal phosphate source instead of tricalcium phosphate.

### Characterization of PSB isolates

The colony characteristics of PSB isolates on Luria-Bertani (LB) agar plates at 37°C were studied, and the growth of NG-33 strain was assessed on LB agar plate with addition of citrate, sucrose, raffinose, sorbitol as the sole energy source, respectively. Isolate NG-33 was stained using a crystal violet-iodine complex to check gram reaction (Coico, 2006). According to the instructions, the biochemical reaction of NG-33 strain was detected by using the MICRO-biochemical identification tube (Hopebio, Qingdao, China) such as the production of hydrogen sulfide, methyl red, pigment, acetylmethyl carbinol and arginine dihydrolase activity.

### PCR amplification and sequencing of 16S rDNA

The DNA was extracted from fresh bacterial culture by using the TaKaRa MiniBEST Bacteria Genomic DNA Extraction Kit. The 16S rDNA was amplified by PCR with the extracted DNA and universal primers (27F: GAGTTTGATCCTGGCTCAG-30 and 1492R-ACCTTGTTACGACTT; Calheiros et al., 2010), where the PCR mix consisted of deoxynucleotides at 200 mM each, 0.25 mM of each primer, 2.5 mM MgCl_2,_ 1 PCR buffer, and 0.2U of Taq DNA polymerase (5 Prime GmbH). The PCR products were purified and then sequenced by an external laboratory (BGI-Shenzhen, Shenzhen, China).

### Identification of *Enterobacter* species

The identification of *Enterobacter* species was based on PCR amplification of 4 specific genes (*dnaA*, *gyrB*, *rpIB*, and *acmP*) with sequence-specific primers (Supplementary Table S1; Miyoshi-Akiyama et al., 2013). The PCR was performed as described above. In addition, the identification also referred to descriptions of the characteristics of mobility and the biochemical reactions of bacteria in Bergey’s Manual of Determinative Bacteriology (Buchanan and Gibbons, 1974).

### Construction of phylogenetic tree

The phylogenetic tree was constructed by using the MEGA 4 program (Tamura et al., 2007) following the neighbor-joining method.

### Analysis of terminal restriction fragment length polymorphisms of 16S rDNA in soil

The total microbial DNA in the rhizosphere soil was isolated by using SoilGen DNA Kit (CWBIO, Beijing, China), quantified by using NanoDrop 2000c spectrophotometer (Thermo Fisher Scientific), and estimated for size through 1% agarose gel electrophoresis. Then, 16S rDNA in soil was amplified by PCR with fluorescence (FAM) dye labeled F-27 primer (6-FAM-AGAGTTTGATCATGGCTCAG-3′) and unlabeled R1492 primer (5′-TAGGGTTACCTTGTTACGACTT-3′). The PCR-amplified DNA was digested overnight by using *Hha* I (Promega, United States) at 37°C (Nithya and Pandian, 2012) in 20 μl reaction system containing 2 μl of the 10× buffer, 17 μl of the purified PCR products and 1 μl of the restriction endonuclease (10 U). The 5 μl of the *Hha* I-digested products were used for electrophoresis on a 1.5% agarose gel. Length of restricted DNA fragments was determined by using an automated ABI DNA sequencer (Model 3,500, Applied Biosystems, United States). The fluorescently labeled 5′-terminal restriction fragments were detected by the GeneMaker 4.0 (Applied Biosystems, United States) and with size markers ranging between 50 and 500 bp, which covered most of the major terminal restriction fragments (T-RFs). T-RFs were calculated as the peak area of the respective T-RF divided by the total peak area of all T-RFs (Sheng et al., 2012). The taxonomic analysis was carried out by using the TRiFLe program (Junier et al., 2008). The statistical analysis was conducted based on complete sample profiles. Several diversity indexes such as Species Richness index (S), Shannon-Weiner index (H) and Pielou’s evenness index (E) were calculated as the literature (Wang et al., 2009).

### Assay of indole-3-acetic acid (IAA)

Indole-3-acetic acid was qualitatively assayed through colorimetry as described by Huang et al. (2013). Briefly, bacteria (NG-33 strain) was cultured at 37°C on shaking cultivation at 180 rpm in LB medium. The bacterial culture was collected at OD_600_ = 0.5.2 ml bacterial culture were taken into EP tube and was centrifuged at 12000 rpm for 2 min, and then 1 ml of supernatant was taken to mix with 1 ml developer Salkowski’s reagent. The mixed solution was incubated in darkness for 20 min at 25°C. The absorbance of mix was measured at 535 nm and the production of bacterial IAA was calculated against the standard curve. The non-inoculated culture was used as the control.

### Assay of pH and organic acids

Bacteria were cultured for 5 days at 37°C by shaking at 150 rpm in NBRIP liquid medium. During culture, the pH of culture liquid was recorded each day with a pH meter equipped with a glass electrode. Meanwhile, the content of organic acids in culture liquid was quantitatively determined. In brief, 1 ml of the culture liquid was transferred to 2 ml tube followed by centrifugation for 5 min at 12,000 rpm. The supernatant was diluted 10 times with distilled water and filtered using 0.22-μm filter membrane. The resulting filtrate was used for assay of organic acid by using the Thermo Scientific Dionex ICS-5000 system (Chi and Huddersman, 2007), which was operated at 30°C of the column temperature in the isocratic mode. For ion exclusion, samples were injected *via* a 25 μl loop, and eluted at a flow rate of 1 ml min^−1^ and a pressure of 2.9 MPa through an IonPac AS11-SC guard column (50 mm × 4 mm) coupled to an IonPac AS11-SC analytical column (250 mm × 4 mm). Peak areas and retention times were recorded and used to calculate chromatographic parameters.

As for content of organic acids in rhizosphere soil, 10 g rhizosphere soil were transferred into a 150 ml conical flask containing 50 ml distilled water, incubated for 60 min by shaking at 200 rpm, and centrifuged at 10000 rpm for 10 min. The supernatant were freeze–dried with a vacuum freeze dryer at −20°C. The lyophilized sample were dissolved with 5 ml distilled water and filtered using 0.22-μm filter membrane. Finally, 25 μl were taken to determine organic acid contents using the Thermo Scientific Dionex ICS-5000 system (Chi and Huddersman, 2007).

### Pot experiment in greenhouse

A pot experiment was conducted in a greenhouse. The pot soils were loamy soil (P sufficient) and sandy soil (P deficit), where P content was defined as high or low (Toth et al., 2014). The loamy soil was the topsoil within 10 cm from Guangxi University’s field station (Guangxi, Nanning), in which total and soluble P contents were 180.3 mg kg^−1^, of 1.12 mg kg^−1^ with a pH of 7.28, respectively. The sandy soil was collected from a sandy site at Nanning in Guangxi, with a low phosphate content (total P of 130 mg kg^−1^; soluble P of 0.79 mg kg^−1^; and pH of 4.62), and tricalcium phosphate (1.2 g kg^−1^ dry soil) was added before use. The pot (6 cm × 8 cm) was filled with 400 g of dry soil, and 30 pots were for each treatment that were designed as inoculation and non-inoculated control groups. During maize planting, 200 ml of 0.25 × Hoagland nutrient solution without phosphorate was added per pot every 3 days.

To prepare the inoculation, a single colony was transferred to 250 ml flasks containing 100 ml LB medium and grown aerobically in a rotating shaker at 200 rpm at 37°C for 10 h. Bacterial suspensions were centrifuged, and the pellets were re-suspended in sterile deionized water. Seeds of maize inbred line Zh58 (90) were soaked for 4 h in bacterial suspensions of 5 × 10^7^ colony-forming units ml^−1^ for inoculation group and in deionized water for control group before sowing. The bacterial suspension (2 ml pot^−1^) was carefully added to the surface soil near the seeds at day 10 and day 20 after sowing while the same volume of deionized water was added for control group.

Growth of maize plants were measured and recoded at 24 days after inoculation, including plant height (from the soil surface to the base of the top leaf with a ruler, dry weights of roots, shoots from roots to the crown, and root–shoot ratio (Fan et al., 2015). A 0.3 g dry and grounded root material was digested in a digestion tube to assay total P concentration by colorimetry (Unicam, Helios Gamma, Cambridge, United Kingdom; Novozamsky et al., 1983; Walinga et al., 1989).

### Measurement of P content in roots and soil

P content in roots were analyzed by colorimetry (Walinga et al., 1989). The soil attached to roots was collected as rhizosphere soil by strongly shaking and then sieved with 20 stainless steel mesh. P content in roots was assayed following the method described by Olsen et al. (1954).

### Measurement of photosynthetic parameter

Photosynthetic rate (*Pn*) and stomatal conductance (*g_s_*) were measured between 9:00 and 11:00 h on clear sunny days by using a Li-6400XT Portable Photosynthetic System with a 6,400-02B LED Red/Blue Light Source (Li-Cor, Lincoln, NE, United States). The uppermost fully expanded leaves in pot plants were selected to measure *Pn* and *g_s_* at the 5-leaf stage.

### Statistical analysis

Statistical difference of data was analyzed by using SPSS 13.0 software (SPSS, Chicago, IL, United States) according to *T*-test at *p* < 0.05 (^*^) or *p* < 0.01 (^**^).

## Results

### Phosphate-solubilizing bacteria isolates

Five bacterial isolates were observed, which exhibited a clear halo by dissolving tricalcium phosphate (TCP) on agar plate (Supplementary Table S2). Their ability to dissolve phosphate was further determined in NBRIP liquid medium, of which P production of one isolate, named NG-33, was up to P 180.7 μg ml^−1^ after 3 days of inoculation.

**Figure 1:**
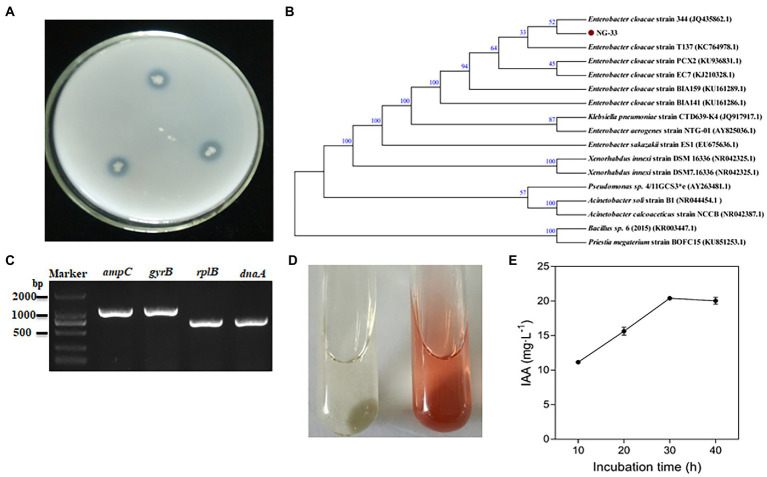
Characteristics of *Enterobacter cloacae* NG-33. **(A)** Bacteria grown for 3 days at 37°C on NBRIP plate culture. **(B)** Neighbor-joining phylogenetic dendrogram based on 16S rDNA sequences showing relationships between the NG-33 strain and their closest taxa. The numbers at the nodes indicate the levels of bootstrap support based on data for 1,000 replicates; only values greater than 50% are presented. The scale bar represents the observed number of changes per nucleotide position. **(C)** The PCR products of four genes (*dnaA*, *gyrB*, *ampC* and *rplB*) of *Enterobacter cloacae* strain NG-33. **(D)** Qualitative assay of IAA production in LB liquid medium. The brown color indicates IAA production (right) after inoculating the NG-33 strain compared to that of the control (left). **(E)** IAA quantitative assay corresponding to the culture duration. NBRIP, National Botanical Research Institute’s Phosphate; IAA, indole-3-acetic acid; LB, Luria-Bertani’s medium (color figure available online).

NG-33 could form colonies of a round, smooth, convex and wet surface, and light yellow and opaque on LB agar plates. Strain NG-33, however, the colonies were ivory colored on the NBRIP agar medium supplemented with 5 g l^−1^ TCP (Figure 1A). The 16S rDNA sequence of NG-33 was 1,420 bp in length (GenBank accession no. KU981056.1). By the phylogenetic analysis, NG-33 appeared to be distinct from those of known species of rhizobia, most closely related to *E. cloacae* strain 344 (GenBank accession no. JQ435862.1; Figure 1B). PCR results (Figure 1C) followed by DNA sequencing and sequence alignment (Supplementary Table S3) showed that GX-33 had four genes (*dnaA*, *gyrB*, *ampC*, and *rplB*) unique to *E. cloacae* subsp. The biochemical reactions of isolate NG-33 were also consistent with *E. cloacae* subsp. (Table 1) according to the description of Bergey’s Manual of Determinative Bacteriology (Buchanan and Gibbons, 1974). Isolate NG-33 should be *E. cloacae* strain NG-33 (GenBank accession no. KU981056.1).

**Table 1:** Biochemical characterization of the *Enterobacter cloacae* NG-33 strain.

Physiological and biochemical tests	Results
Motility	+
Arginine dihydrolase test	+
Acetylmethyl carbinol test	+
Methyl red test	−
Hydrogen sulfide test	−
Citrate utilization test	+
Gelatin hydrolysis test	−
Sucrose utilization test	+
Raffinose utilization test	+
Sorbitol utilization test	+
Pigment test	−

+, tested positive/utilized as substrate; −, tested negative/not utilized as substrate.

### Indole-3-acetic acid production and phosphate-solubilizing characterization of NG-33

**Figure 2:**
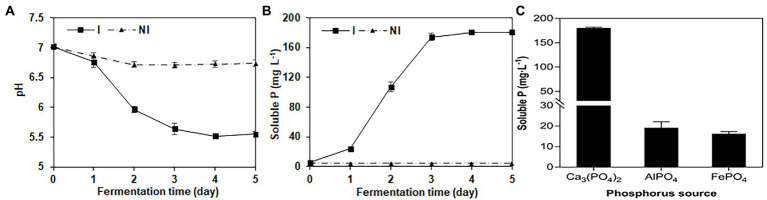
Changes of pH value **(A)** and soluble P **(B)** in National Botanical Research Institute’s Phosphate medium (NBRIP) liquid broths with the extension of the fermentation time, and soluble P in the NBRIP liquid broths supplemented with the various phosphorus sources **(C)** after the inoculation with NG-33.

NG-33 could produce IAA (Figure 1D) and showed a production of 20.4 mg L^−1^ (Figure 1E). During fermentation in NBRIP liquid medium under NG-33 inoculation, pH of fermentation broth decreased from initial 7.0–5.6 after 3 days of fermentation (Figure 2A), and soluble P concentration was up to 180.7 μg ml^−1^ after 3 days of fermentation (Figure 2B). When calcium phosphate in the NBRIP solution was replaced by aluminum phosphate or iron phosphate, of soluble P in fermentation broth was 16.3–19.2 μg ml^−1^ (Figure 2C).

### Organic acid production by NG-33

**Figure 3:**
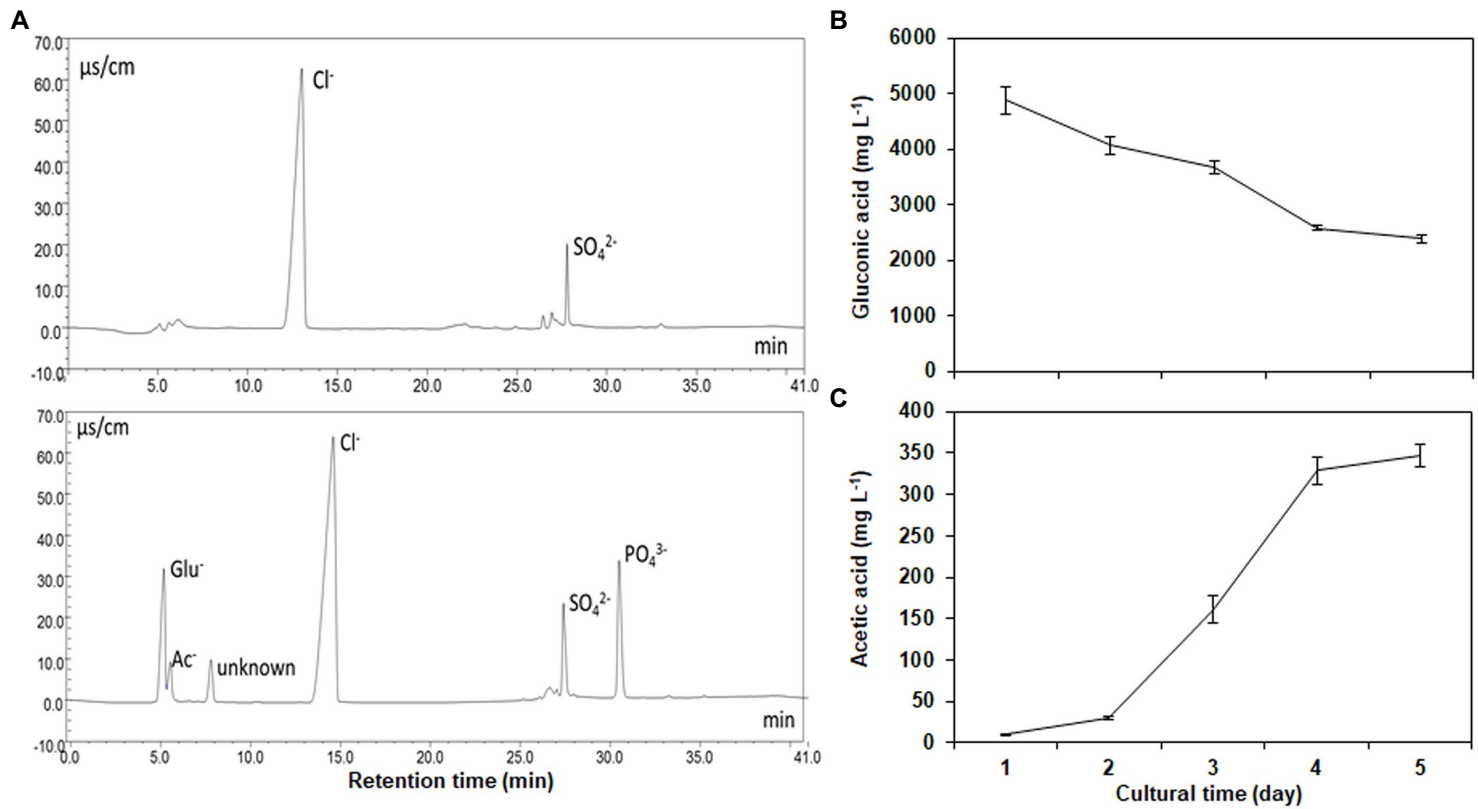
Separation of organic acids and inorganic anions using an IonPac AS11-SC analytical column **(A)**, and the production of gluconic acid **(B)** and acetic acid **(C)** by inoculating the strain NG-33 on the NBRIP liquid medium.

Gluconic acid, acetic acid, and an unknown acid were identified in the supernatants of the cultural broths, respectively (Figure 3A). Strain NG-33 produced a maximum gluconic acid (4,881 mg l^−1^) one day after culture at 37°C and then decreased to 2,392 mg l^−1^ after 5 days (Figure 3B). Acetic acid produced by NG-33 gradually accumulated in the culture liquid and reached 346 mg l^−1^ after 5 days of fermentation (Figure 3C).

### Plant growth-promoting traits of NG-33

**Figure 4:**
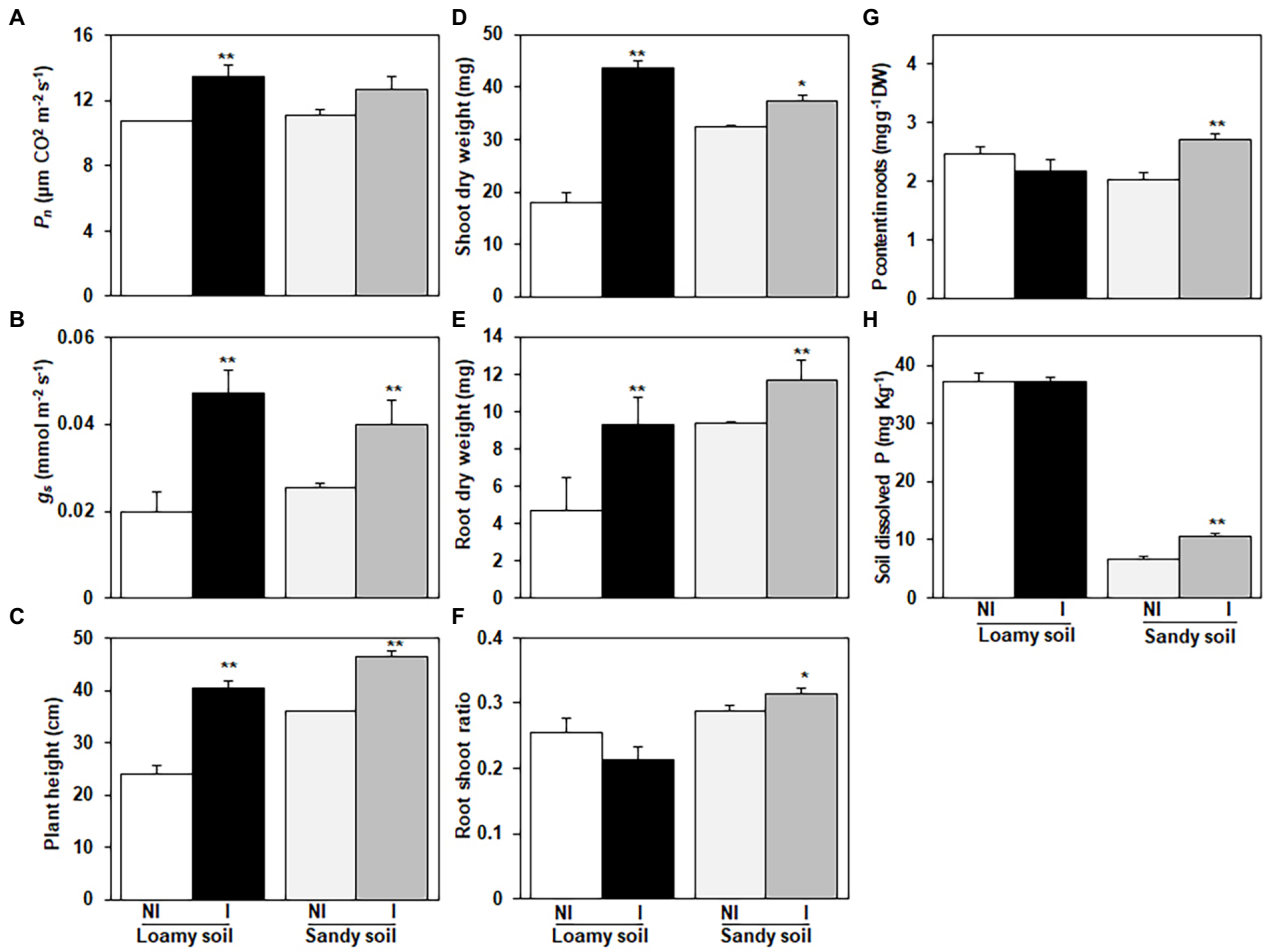
Effects of *Enterobacter cloacae* NG-33 strain inoculation on leaf net photosynthetic rate (*P*_n_); **(A)** and stomatal conductance (*G_s_*); **(B)**, plant height **(C)**, shoot dry weight **(D)**, root dry weight **(E)**, root shoot ratio **(F)**, P content in roots of maize seedlings (Zh58) **(G)** and soil dissolved P content after harvest **(H)**. Data are the mean and ± SE of four replicates for inoculated (I) and non-inoculated (NI) plants under loamy soil (subtropical) and sandy soil (phosphorus-deficient) with supplemental application of tricalcium phosphate. The difference between the means is significant at *p* < 0.05 (^*^) and *p* < 0.01 (^**^) level.

Net photosynthetic rate was 25.6% higher in the inoculated plants than in control plants when grown in loamy soil. Similarly, when grown in sandy soil, with additional TCP, the inoculated plants significantly increased their net photosynthetic rate by 14.2% than control plants (Figure 4A). Inoculation with NG-33 increased the *g*_s_ by 135.8% in loamy soil and 58.0% in sandy soil with additional TCP compared to control plants (Figure 4B). Plant height was significantly higher in plants inoculated NG-33 than in non-inoculated plants, in both the control and with additional TCP. Compared to the control plants, the addition of TCP had higher plants, as the inoculated plants had 68.9% higher plant height in TCP and 29.5% higher plant height in control treatment (Figure 4C).

The inoculation of NG-33 had a positive and promoting effect on maize seedlings grown in both loamy and sandy soil. For the loamy soil, the NG-33 inoculation induced a 140.8% increase (*p* < 0.01) in shoot dry weight and 97.0% increase (*p* < 0.01) in root dry weight at the 5-leaf stage (Figures 4D,E). In sandy soil with the supplemental application of TCP, the NG-33 inoculation significantly increased the shoot and root dry weight by 14.9 and 24.9% (*p* < 0.05, *p* < 0.01), respectively (Figures 4D,E). Furthermore, our results showed that the NG-33 inoculation significantly increased root/shoot ratios in sandy soil but markedly decreased in loamy soil (*p* < 0.05; Figure 4F).

The inoculation of NG-33 had a significant effect on root P content under sandy soil with supplemental application of TCP, suggesting 33.6% higher P content in maize root compared to non-inoculated plants (*p* < 0.01; Figure 4G). However, under loamy soil the P content of maize root in inoculated and non-inoculated were statistically similar. After harvest, the soil dissolved P content in inoculated soil was 58.6% higher compared to non-inoculated soil in sandy soil with the supplemental application of TCP (*p* < 0.01; Figure 4H). In contrast, the inoculation did not significantly change soil dissolved P content in the loamy soil (Figure 4H).

NG-33 inoculation significantly increased malic acid, tartaric acid, and oxalic acid, while acetic acid and citric acid were only found in NG-33 inoculated pots in both soil types (Table 2). The malic acid content was the highest in sandy soil compared to loamy soil, and slightly increased due to NG-33 inoculation. The further demonstrated that the inoculated soil increased tartaric acid and oxalic acid by 8- and 3-fold in loamy soil, and by 10 and 11-fold in sandy soil with additional application of TCP. In inoculation soil, the content of acetic acid and citric acid were 49.3 and 7.9 mg kg^−1^ in the loamy soil, and 35.2 and 8.8 mg kg^−1^ in sandy soil with supplemental TCP application, respectively.

**Table 2:** Effect of *Enterobacter cloacae* NG-33 strain inoculation on organic acid composition under loamy and sandy soil with supplemental tricalcium phosphate (TCP) application.

Soil treatments		Malic acid	Tartaric acid	Oxalic acid	Acetic acid	Citric acid
Loamy soil	NI	138.7 ± 17.3	1.2 ± 1	1.89 ± 0.0	–	–
	I	153.7 ± 24.7	9.6 ± 5.1	6.2 ± 0.7	49.3 ± 20.7	7.9 ± 1.5
Sandy soil	NI	334.4 ± 19.2	2.0 ± 2.3	1.4 ± 0.1	–	–
	I	337.5 ± 3 8.1	19.2 ± 4.8	15.3 ± 6.6	35.2 ± 24.7	8.8 ± 0.4

Types (mg kg^−1^).

– showed that the acetic acid/citric acid was not found in the soil without the E. cloacae NG-33 strain inoculation. NI, without the inoculation; I, inoculation.

### Bacterial community structure

**Figure 5:**
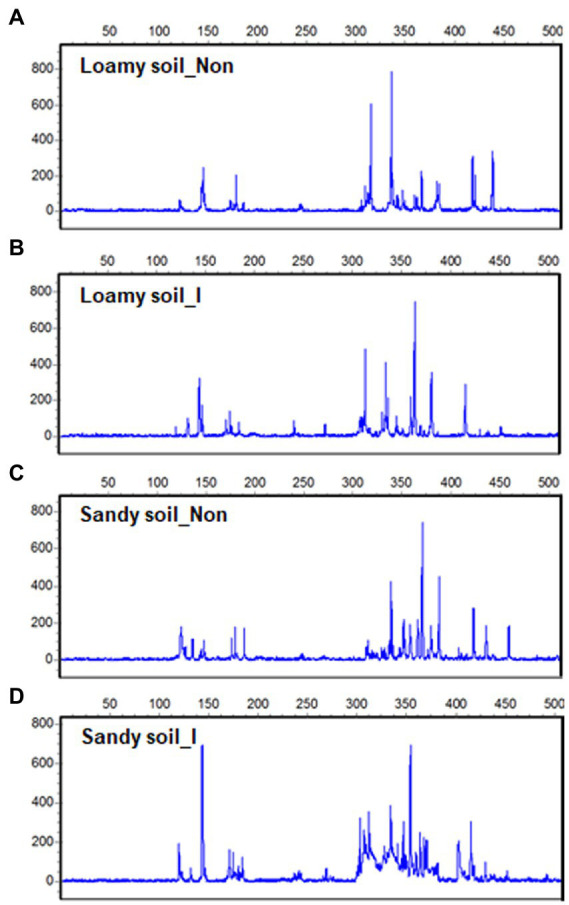
Terminal restriction fragment length polymorphisms (T-RFLP) in soil bacterial 16S rDNA from the rhizosphere of maize seedlings with inoculation and non-inoculation under loamy and sandy soil with supplemental application of tricalcium phosphate (TCP). One out of 3T-RFLP profiles was shown for loamy soil with non-inoculation **(A)** and inoculation **(B)**, TCP soil with non-inoculation **(C)** and inoculation **(D)**.

After maize harvest, we obtained 12 T-RFLP profiles out of soil DNAs extracted from the rhizosphere of the inoculated and non-inoculated plants under the loamy and sandy soil. At the same time, other 12 T-RFLP profiles out of soil DNAs were extracted from the inoculated pots and non-inoculated pots without growing plants. Most T-RFs were detected between 50 and 500 bp size range (Figures 5A–D). After aligning T-RFs, 119 different T-RFs were identified with *Hha* I enzyme, and 27 fragments showed highly repetitive.

**Figure 6:**
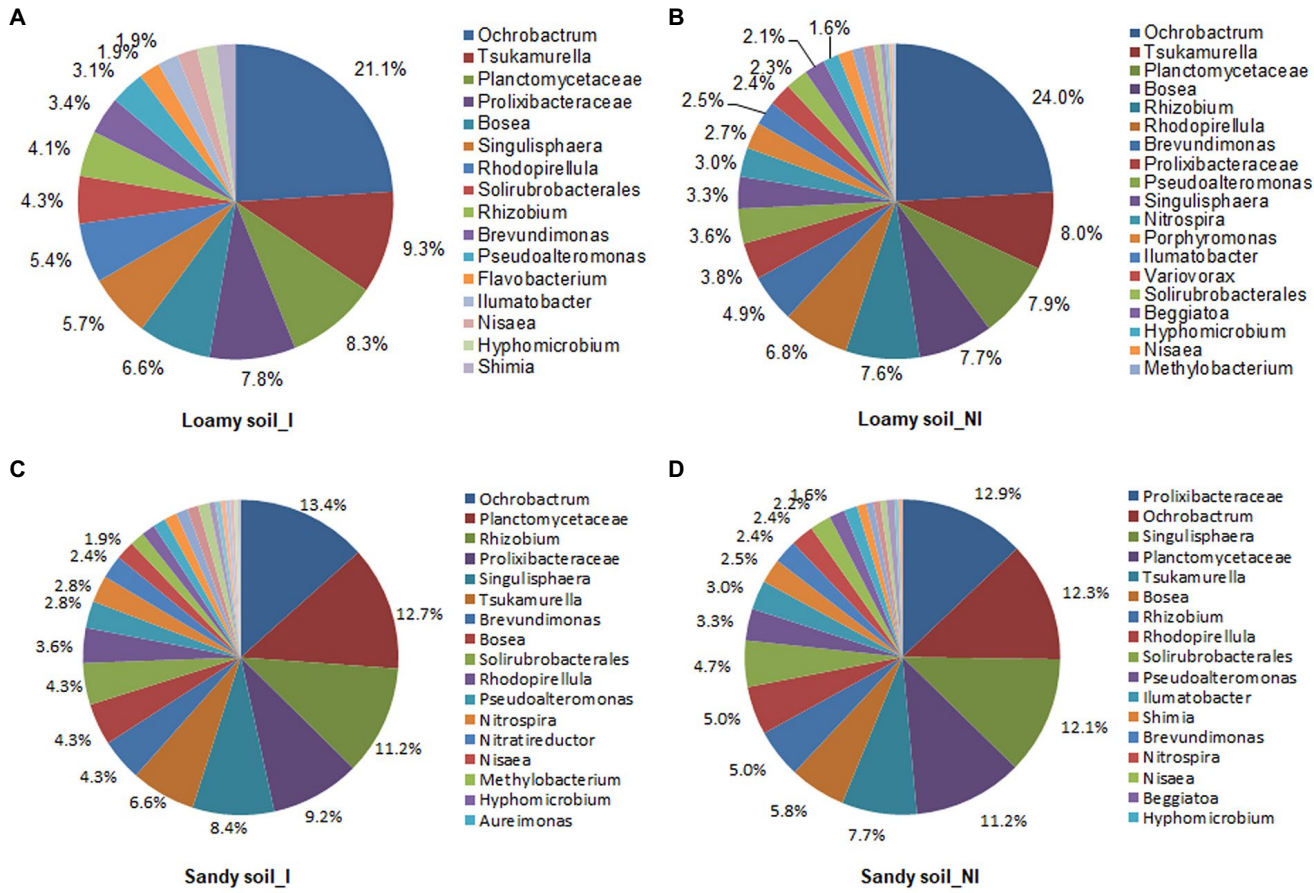
Schematic representation of bacterial communities in loamy and sandy soil with and without inoculation of *Enterobacter cloacae* NG-33 strain. The differences of bacterial communities composition among the inoculated **(A,C)** and non-inoculated **(B,D)** in loamy and sandy soil with the supplemental application of TCP. I, inoculation; NI, non-inoculation.

Based on T-RFLP profiles data, total of 36 microbes were identified in the inoculated and non-inoculated treatments under two different soil types. The NG-33 inoculation in loamy soil had greater numbers of microbes (34) compared to non-inoculated soil (25), suggesting 36% higher soil microbes in inoculated soil. Similarly, 28 microbes were observed in inoculated soil and 24 in non-inoculated soil under sandy soil with a supplemental application of TCP. Different soil types and NG-33 inoculation had a different effect on dominant soil microbes. *Ochrobactrum* (21.1 and 24.0%), *Tsukamurella* (9.3 and 8.0%), and *Planctomycetaceae* (8.3 and 7.9%) were the most abundant soil microbes in loamy soil with and without inoculation of NG-33, respectively (Figures 6A,B). The abundances of *Prolixibacteraceae* and *Singulisphaera* increased, whereas the abundances of *Bosea* and *Rhizobium* greatly decreased in loamy soil with NG-33 inoculation compared to non-inoculated soil. In addition, the *Ochrobactrum* decreased by 12.1%, whereas soil inoculation increased the abundance for *Tsukamurella* by 16.3% and for *Planctomycetaceae* by 5.1% compared to those of non-inoculated soil (Figures 6A,B). In non-inoculated sandy soil, the dominant species were *Prolixibacteraceae* (12.9%), *Ochrobactrum* (12.3%), and *Singulisphaera* (12.1%), while *Ochrobactrum* (13.4%), *Planctomycetaceae* (12.7%), and *Rhizobium* (11.2%) were dominant species in NG-33 inoculated sandy soil (Figures 6C,D). Compared to non-inoculated sandy soil, NG-33 inoculation increased the abundance in *Ochrobactrum* by 8.94%, in *Planctomycetaceae* by 13.39%, and in *Rhizobium* by 124%, whereas, there was a decrease in the abundance of *Prolixibacteraceae* by 28.68% and *Singulisphaera* by 30.58% (Figures 6C,D).

**Figure 7:**
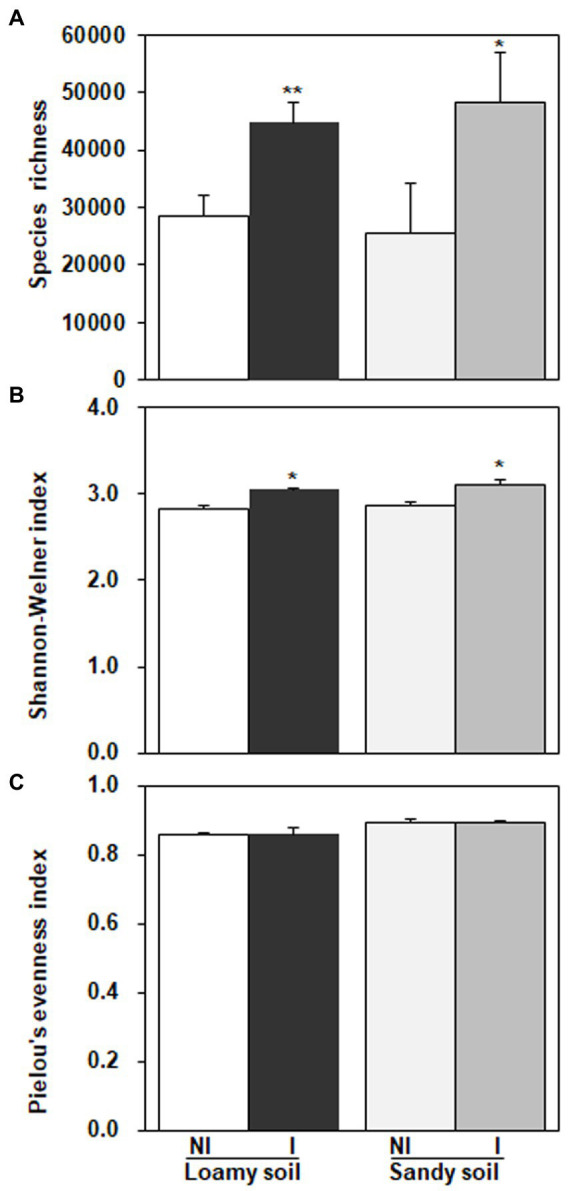
Diversity indexes obtained from the restriction enzyme *Hha I* in loamy and sandy soil with and without inoculation of *Enterobacter cloacae* NG-33 strain. **(A)** Species richness, **(B)** Shannon-Weiner index, **(C)** Pielou’s evenness index. The difference between the means is significant at *p* < 0.05 (^*^) and *p* < 0.01 (^**^) level.

**Figure 8:**
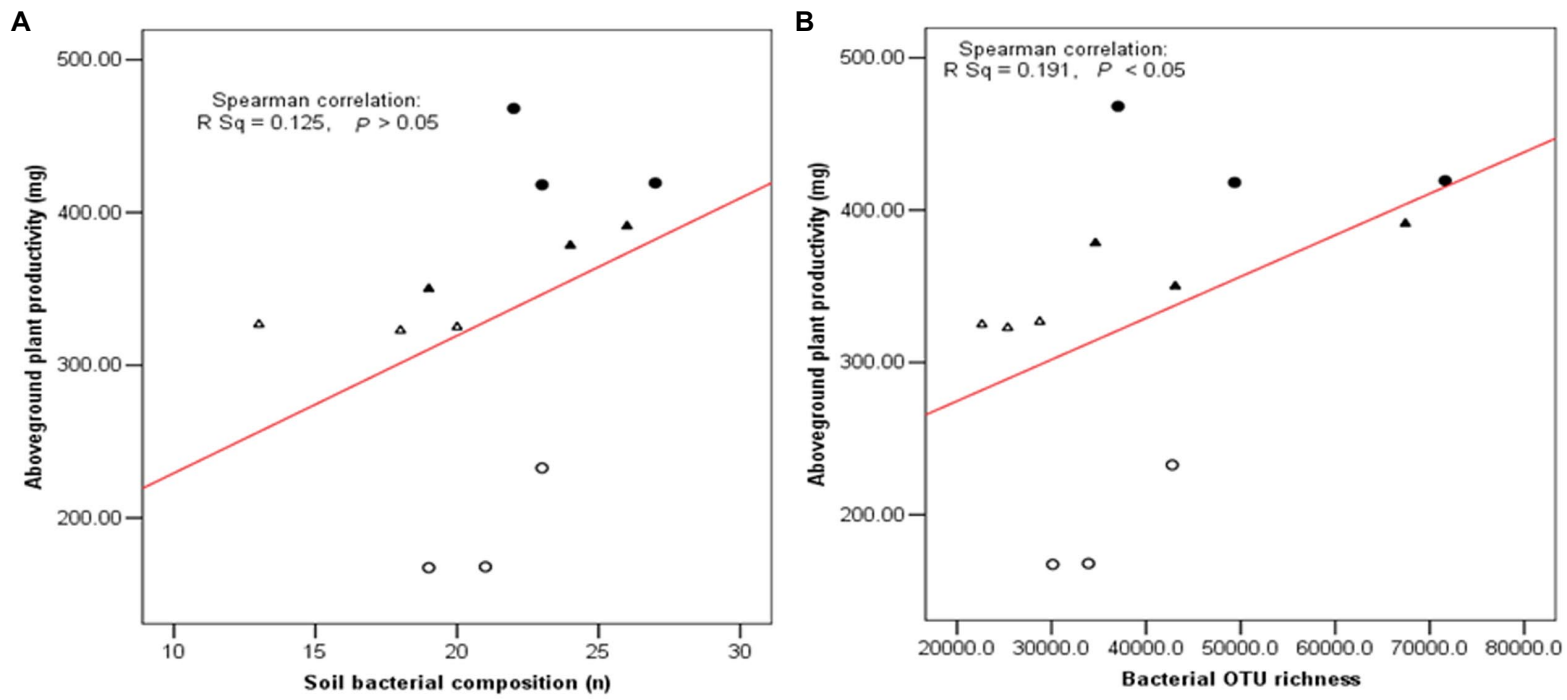
Relationship between plant biomass and soil bacterial composition **(A)** and bacterial OTU richness **(B)** in soil. Filled triangle (▲) and Triangle (∆) indicated the inoculation with NG-33 and non-inoculation in the sand soil with supplement of TCP, and filled cycle (●) and cycle (○) indicated the inoculation with NG-33 and non-culation in the loamy soil.

As shown in Figure 7, three diversity indexes obtained from the restricted enzyme *Hha* I. The species richness, Shannon-Weiner index, and Pielou’s evenness index were used to signify the diversity. The species richness and Shannon-Weiner index across the NG-33 inoculation treatment were higher both in sandy soil supplemented with TCP and loamy soil (Figure 7). Furthermore, these results suggest that NG-33 inoculation significantly increased species richness and Shannon-Weiner index in both soils compared to non-inoculated soil. Whereas Pielou’s evenness index showed no significant differences between inoculated and non-inoculated soil. The regressions analysis showed that the aboveground plant productivity was not significantly affected by soil bacterial composition (*R*^2^ = 0.125, *p* > 0.05; Figure 8A), but there was a significant positive correlation between the aboveground plant productivity and bacterial OTU richness (*R*^2^ = 0.191, *p* < 0.05; Figure 8B).

## Discussion

### Phosphate-solubilizing capacity of NG-33

NG-33 strain showed a great potential for converting insoluble TCP to soluble phosphate (180.7 μg ml^−1^) with a decrease in the pH of the medium (5.6) after 3 days of inoculation, and released soluble P from metal inorganic sources Al-P and Fe-P by 19.2 and 16.3 μg ml^−1^, respectively. This suggested a strong capacity of strain NG-33 to solubilize P from inorganic pools. This ability is superior to those of previous study (Shahid et al., 2012), which demonstrated that the inoculation of *E. cloacae* Fs-11 had the ability to solubilize 43.5 μg ml^−1^ of TCP in to soluble P. Although *E. cloacae* LCR1 and LCR2 exhibited a high level of phosphate solubilization (568–642 μg ml^−1^) capacity in the NBRIP medium (Kumar et al., 2010), its role in plant growth-promoting is still unknown. The current study found that the *E. cloacae* NG-33 can produce indole acetic acid in addition to release soluble P from the insoluble inorganic pools, likely TCP, Fe-P and Al-P. These finding are consistent with those of Shankar et al. (2011), which discovered that the *E. cloacae* GS1 inoculation promoted rice growth and produced indole acetic acid, however not related to phosphate solubilization.

### Correlation of organic acid production with phosphate solubilization by NG-33

Most mechanisms of phosphate solubilization are related to the production of organic acids by PSB, which lowers the pH in the surrounding soil (Wei et al., 2010). Two important metabolites produced by *E. cloacae* NG-33 strain identified in the current study were gluconic and acetic acids. A third unknown acid was also discovered in the NBRIP medium broths. Yahya et al. (2021) showed that P-solubilizing activity was predominantly associated with the production of gluconic acid and acetic acid, causing a decrease in pH (up to 3.5). There was a positive and strong relationship between solubilizing phosphate capacity and gluconic and acetic acid concentrations. The relationship is supported by other PSB strains, i.e., *Enterobacter* sp. ZW32 and *Ochrobactrum* sp. SSR, which shows a strong P-solubilizing capabilities by the production of organic acids (Yahya et al., 2021). The *E. cloacae* Fs-11 could also secrete gluconic acid by 16.64–117 μg ml^−1^ and increase total phosphorus contents in inoculated plants (Shahid et al., 2012), while *E. cloacae* NG-33 could secrete more gluconic and acetic acids, suggesting a potential capacity of promoting plant growth under soil inoculation.

### Plant growth-promoting effects by NG-33

PSB inoculation has positive effects on P availability of rhizosphere soil, and improves plant growth (Wu et al., 2019). The NG-33 strain inoculation have a remarkable effect on leaf photosynthetic rate and markedly increase plant height by 30–69%, shoot by 15–141%, and root biomass by 25–97% both in loamy and sandy soil, which is better in promoting effect of maize than other results reported by Ateş et al. (2022). Likely because the inoculation NG-33 increased plant nutrition availability by converting insoluble P sources. Furthermore, the results demonstrated that the increase in shoot and root biomass were associated with an increase in *P*_n_ and *g_s_*. This is supported by other PSB strains where the application of bacterial consortium and phosphate-solubilizing fungus augmented plant photosynthetic ability and *g_s_* in both normal and salt stress (Khanghahi et al., 2021; Zai et al., 2021). This suggest that the *E. cloacae* NG-33 strain had the potential to be developed as a bio-fertilizer in the subtropical zone and phosphorate limited soils. In addition, the inoculation of *E. cloacae* NG-33 strain into sandy soils dramatically increased the soluble phosphate could be mainly attributed to the more production of organic acids (i.e., tartaric acid, oxalic acid, acetic acid, and citric acid) into the rhizosphere to solubilize and release the available P from insoluble P pools. Microbes can solubilize inorganic phosphate compounds in the rhizosphere by releasing a variety of organic compounds into the environment (Scervino et al., 2010; Xie et al., 2021). However, the inoculation of NG-33 strain also increased leaf *P*_n_ and plant biomass in loamy soil without the supplemental TCP, likely due to the high production of IAA (20.4 μg ml^−1^) by NG-33. This was consistent with the IAA (15 μg ml^−1^) produced by inoculation of *E. cloacae* GS1 to promote rice growth (Shankar et al., 2011). Thus, plant development is boosted by NG-33 inoculation because it can produce significant concentrations of indole acetic acid in the soil and liberate soluble P from the dissoluble phosphate pools.

### Increases in P content in roots and dissolved P in sandy soil by the inoculated NG-33

Bashan et al. (2013) reported that an isolate must be further evaluated to test whether it directly contributes to plant P before it can be declared as PSB. The NG-33 strain obviously contributes greatly to the accumulation of P content nourishment in roots, but can also enhance the dissolved P content in sandy soil with supplemental TCP, which could directly result in higher plant growth and photosynthetic production. However, the inoculation of the NG-33 strain had no significant effect on root P content and dissolved P in loamy soil, even though the plant biomass increased significantly after inoculation. The plant growth promoting compounds, such as IAA and ACC-deaminase activity are likely to the key sources and responsible for the plant growth and development (Pereira and Castro, 2014; Fan et al., 2015). These findings suggest that the NG-33 strain has a unique mechanism for promoting plant development in different soils.

### Optimization of soil bacterial community richness by NG-33

Bacteria are the most abundant and diverse, and they play multiple indispensable roles in soil. Any modifications in the microbial community caused by land use change might contribute to changes in ecosystem function and maintenance of soil quality (Konopka, 2009). The inoculation of *E. cloacae* NG-33 strain significantly increased the diversities of soil microbial communities (Figure 7), which affects the variations of dominant microbes and the composition of microbial communities. Several studies showed that microbial inoculation improved plant growth and crop production, probably because of increasing plant nutrients uptake, sustaining environmental health and soil productivity, and improving plant hormones level (cytokinin and IAA; Liu et al., 2013; Fan et al., 2015; Anzuay et al., 2017). The present study showed that soil inoculation with NG-33 strain altered microbial community composition, resulting in a significantly higher soil microbial species richness and Shannon-Weiner index, but the inoculation did not change pielous evenness index. The addition of appropriate fertilization can also increase microbial activity and species richness, but slightly decrease in species evenness (Ali Hassan and Saud, 2020). However, the inoculated soil caused the rebalance of the microbial community and a novel community structure produced more organic acids (i.e., tartaric acid, acetic acid, citric acid, and chlorogenic acid) owing to the enhancement of bacterial community richness. Soil bacterial diversity are closely associated with soil nutrient levels and soil productivity (Wu et al., 2015). In addition, the regression analysis showed that the aboveground biomass was significantly positively correlated with bacterial OTU richness independent of soil inoculation.

## Conclusion

Novel PSB *E. cloacae* NG-33 strain possessed a strong phosphate-solubilizing ability associated mainly with a high production of gluconic and acetic acid. Soil inoculated with *E. cloacae* NG-33 strain increased maize biomass production in loamy soil and sandy soil with supplemental TCP application, which were related to the improvement of soil quality (soil P content) caused by its phosphate-solubilizing ability, and its increasing diversity of soil microbe community in inoculated soil.

## Data availability statement

The datasets presented in this study can be found in online repositories. The names of the repository/repositories and accession number(s) can be found in the article/Supplementary material.

## Author contributions

XF conceived and designed the study, and supervised the project, wrote and revised the manuscript. XC performed most of the experiments. CY performed part of the experiments and prepared the Figures. YL participated in data analysis and revised manuscript. JP reviewed the manuscript and edited its structure and presentation. All authors contributed to the article and approved the submitted version.

## Funding

The study is funded by Guangxi Key Research and Development Program (GuikeAB21238005).

## Conflict of interest

The authors declare that the research was conducted in the absence of any commercial or financial relationships that could be construed as a potential conflict of interest.

## Publisher’s note

All claims expressed in this article are solely those of the authors and do not necessarily represent those of their affiliated organizations, or those of the publisher, the editors and the reviewers. Any product that may be evaluated in this article, or claim that may be made by its manufacturer, is not guaranteed or endorsed by the publisher.
