# Supplementary material for: An Enterobacter cloacae strain NG-33 that can solubilize phosphate and promote maize growth
Source: Front Microbiol. 2022 Nov 10;13:1047313. doi: 10.3389/fmicb.2022.1047313 (PMC9685795; doi:10.3389/fmicb.2022.1047313)
Supplement: Supplementary file 1 [file Data_Sheet_1.docx]

| Primer name | Sequence 5′ → 3′ |
| --- | --- |
| acmP-f (ECA) | CCCTTTGCTGCGCCCTGC |
| acmP-r (ECB) | TGCCGCCTCAACGCGTGC |
| gyrB-f | TCGACGAAGCGCTCGCGGGTCACTGTAA |
| gyrB-r | GCAGAACCGCCCGCGGAGTCCCCTTCCA |
| rplB-f | GTAAACCGACATCTCCGGGTCGTCGCCA |
| rplB-r | ACCTTTGGTCTGAACGCCCCACGGAGTT |
| dnaA-f | AYAACCCGCTGTTCCTBTATGGCGGCAC |
| dnaA-r | KGCCAGCGCCATCGCCATCTGACGCGG |

Table S1. Identification primers of *E. cloacae*

| Isolates number | Phosphate-Solubilizing ring diameter (mm) | Isolates diameter  (mm) | Phosphate-Solubilizing ring /isolates diameter |
| --- | --- | --- | --- |
| NG-12 | 8.3 | 6.2 | 1.34 |
| NG15 | 7.6 | 4.7 | 1.62 |
| NG-33 | 12.4 | 4.1 | 3.02 |
| NG-43 | 44 | 2.4 | 1.83 |
| NG-62 | 3.7 | 1.8 | 2.06 |

Table S2 Comparative analysis of phosphate solubilizing ability of selected target isolates

| Table S3. Sequences of four housekeeping genes in *E. cloacae* NG33 and its significant alignment by BLASTN on NCBI | | | |
| --- | --- | --- | --- |
| Housekeeping genes | Gene sequences in *E. cloacae* NG33 | Alignment to strain name (GenBank accession no.) | Identities |
| ***dnaA*** | >_dnaA PCR  TGGCGGCACGGGTCTCGGTAAAACGCACCTGCTGCATGCGGTGGGCAACG  GCATTATGGCGCGTAAGCCCAATGCCAAAGTGGTGTATATGCACTCCGAA  CGCTTCGTTCAGGACATGGTAAAAGCCCTGCAAAACAATGCGATCGAAGA  GTTTAAACGCTACTACCGTTCCGTTGATGCGCTGCTGATCGATGACATTC  AGTTCTTTGCCAATAAAGAACGATCGCAGGAAGAGTTTTTCCATACCTTT  AACGCCCTGCTGGAAGGCAATCAGCAGATCATTCTGACCTCGGATCGTTA  TCCAAAAGAGATCAACGGCGTTGAAGATCGTCTCAAATCCCGCTTCGGCT  GGGGCCTGACCGTGGCGATCGAGCCGCCGGAGCTGGAAACCCGCGTCGCG  ATCCTGATGAAAAAAGCCGACGAGAACGACATTCGCCTGCCGGGTGAAGT  GGCGTTCTTCATTGCCAAGCGCCTGCGTTCCAACGTGCGTGAGCTGGAAG  GGGCGCTGAACCGCGTTATCGCCAACGCCAACTTCACCGGTCGGGCGATC  ACCATCGATTTTGTGCGTGAAGCGCTGCGCGATTTATTGGCTTTGCAGGA  AAAACTGGTCACTATCGACAATATTCAGAAGACGGTGGCTGAGTACTACA  AGATCAAAGTGGCAGATTTACTGTCTAAACGTCGTTCCCGCTCGGTGGCG  CGTCCGCGTCAGAT | *E. cloacae* strain 58983CZ (CP085734.1) | 100% |
|  |  | *E. cloacae* isolate 1015 (OW849356.1) | 100% |
|  |  | *E. cloacae* isolate 662 (OW969620.1) | 100% |
|  |  | *E. cloacae* STN0717-60 (AP022509.1) | 100% |
| ***gyrB*** | >_gyrB PCR  CTCCGTGTCCGTTACCGATGACGGTCGTGGCATCCCAACCGGTATTCACC  CGGAAGAGGGCGTATCTGCTGCGGAAGTGATCATGACCGTTCTGCACGCA  GGCGGTAAGTTCGATGATAACTCCTATAAAGTGTCCGGTGGTCTGCACGG  CGTAGGCGTATCCGTCGTAAACGCCCTGTCGCAGAAGCTGGAGCTGGTTA  TCCAGCGCGAAGGCAAAATTCACCGTCAGATCTACCAGCACGGCGTGCCT  GAAGCGCCGCTGGCCGTCACGGGTGATACCGAGAAAACCGGTACCATGGT  GCGTTTCTGGCCGAGCCTTGAAACCTTCACCAACGTCACCGAATTCGAGT  ACGACATTCTGGCGAAACGCCTGCGTGAACTGTCGTTCCTGAACTCCGGC  GTGTCGATTCGTCTGCGCGACAAACGCGACAACAAAGAAGACCACTTCCA  TTACGAAGGTGGTATCAAGGCGTTCGTTGAGTATCTGAACAAGAACAAAA  CGCCAATTCACCCGAATATCTTCTACTTCTCTACTGAAAAAGACGGTATC  GGTGTGGAAGTGGCCTTGCAGTGGAACGACGGTTTCCAGGAAAACATCTA  CTGCTTCACCAACAACATTCCACAACGCGATGGCGGTACGCACCTGGCGG  GCTTCCGCGCGGCGATGACCCGAACCCTGAACGCCTACATGGACAAAGAA  GGCTACAGCAAAAAAGCGAAAGTCAGCGCCACCGGTGACGATGCCCGTGA  AGGCCTGATTGCCGTGGTCTCCGTGAAGGTGCCGGATCCGAAGTTCTCCT  CACAGACCAAAGACAAGCTGGTCTCTTCTGAGGTGAAATCGGCGGTTGAA  CAGCAGATGAACGAACTGCTGAGCGAATACCTGCTGGAAAACCCGTCCGA  CGCGAAAATCGTGGTGGGTAAAATTATCGATGCGGCGCGTGCCCGTGAAG  CGGCGCGTAAAGCGCGTGAAATGACCCGTCGTAAAGGCGCGCTGGACCTG  GCAGGCCTGCCGGGCAAACTGGCTGACTGTCAGGAACGCGA | *E. cloacae* isolate 133  ([OW9681](https://www.ncbi.nlm.nih.gov/nucleotide/OW968086.1?report=genbank&log$=nucltop&blast_rank=28&RID=GTFW0RPX016)39.1) | 99.14% |
|  |  | *E. cloacae* complex sp. strain AR_0163  (CP021749.1) | 99.04% |
|  |  | *E. cloacae* isolate 662 (OW969620.1) | 98.94% |
|  |  | *E. cloacae* strain 58983CZ (CP085734.1) | 98.94% |
|  |  | *E. cloacae* STN0717-60 (AP022509.1) | 98.94% |
| ***acmP*** | >_acmP PCR  CTCGCCACGCCAGTGTCAGAAAAACAGCTGGCGGAGGTGGTCGCGAATAC  GGTTACCCCGCTGATGAAAGCCCAGTCTGTTCCAGGCATGGCGGTGGCCG  TTATTTATCAGGGAAAACCGCACTATTACACGTTTGGCAAGGCCGATATC  GCGGCGAATAAACCCGTTACGCCTCAGACCCTGTTCGAGCTGGGTTCTAT  AAGTAAAACCTTCACCGGCGTTTTAGGTGGGGATGCCATTGCTCGCGGTG  AAATTTCGCTGGACGATCCGGTGACCAGATACTGGCCACAGCTGACGGGC  AAGCAGTGGCAGGGTATTCGTATGCTGGATCTCGCCACCTACACCGCTGG  CGGCCTGCCGCTACAGGTACCGGATGAGGTCACGGATAACGCCTCCCTGC  TGCGCTTTTATCAAAACTGGCAGCCGCAGTGGAAGCCTGGCACAACGCGT  CTTTACGCCAACGCCAGCATCGGTCTTTTTGGTGCGCTGGCGGTCAAACC  TTCTGGCATGCCCTATGAGCAGGCCATGACGACGCGGGTCCTTAAGCCGC  TCAAGCTGGACCATACCTGGATTAACGTGCCGAAAGCGGAAGAGGCGCAT  TACGCCTGGGGCTATCGTGACGGTAAAGCGGTGCGCGTTTCGCCGGGTAT  GCTGGATGCACAAGCCTATGGCGTGAAAACCAACGTGCAGGATATGGCGA  ACTGGGTCATGGCAAACATGGCGCCGGAGAACGTTGCTGATGCCTCACTT  AAGCAGGGCATCGCGCTGGCGCAGTCGCGCTACTGGCGTATCGGGTCAAT  GTATCAGGGTCTGGGCTGGGAGATGCTCAACTGGCCCGTGGAGGCCAACA  CGGTGGTCGAGGGCAGCGACAGTAAGGTAGCGCTGGCGCCGTTGCCCGTG  GCAGAAGTGAATCCACCGGCTCCCCCGGTCAAAGCGTCCTGGGTCCATAA  AACGGGCTCTACTGGCGGGTTTGGCAGCTACGTGGCCTTTATTCCTGAAA  AGCAGATCGGTA | *E. cloacae* strain 4-52 class (KJ135993.1) | 99.9% |
|  |  | *E. cloacae* MS22.1 (NG_048602.1) | 99.8% |
|  |  | *E. cloacae* 919729 (NG_048620.1) | 99.8% |
|  |  | NG_050703.1 (*E. cloacae* 28132) | 99.8% |
|  |  | *E. cloacae* 35415 (NG_050706.1) | 99.8% |
| ***rpIB*** | >_rplB PCR  TCCGGGTCGTCGCCACGTAGTTAAAGTGGTTAACCCTGAGCTGCACAAGG  GCAAACCTTTTGCTCCGTTGCTGGAAAAAAACAGCAAATCCGGTGGTCGT  AACAACAATGGCCGTATCACCACTCGTCACATCGGTGGTGGCCACAAGCA  GGCTTATCGTATTGTTGACTTCAAACGCAACAAAGACGGTATCCCAGCAG  TTGTTGAGCGTCTTGAGTACGATCCGAACCGTTCCGCGAACATCGCGCTG  GTTCTGTACAAAGATGGCGAACGCCGTTACATCCTGGCCCCTAAAGGCCT  GAAAGCTGGCGACCAGATTCAGTCTGGCGTTGATGCTGCAATCAAAGCAG  GCAACACCCTGCCGATGCGCAATATCCCGGTTGGTTCTACCGTTCATAAC  GTAGAAATGAAACCAGGTAAAGGCGGTCAGCTGGCGCGTTCCGCGGGTAC  TTACGTTCAGATCGTTGCGCGTGACGGTGCTTATGTCACCCTGCGTCTGC  GTTCTGGTGAAATGCGTAAAGTCGAAGCAGACTGCCGCGCTACTCTGGGC  GAAGTTGGCAATGCTGAGCATATGCTGCGCGTTCTGGGTAAAGCTGGTGC  TGCACGCTGGCGTGGTGTTCGTCCTACCGTTCGCGGTACTGCGATGAACC  CAGTCGACCACCCACATGGTGGTGGTGAAGGTCGTAACTTTGGTAAGCAC  CCGGTAACTCCGTGGGGCGTT | *E. cloacae* isolate 93 (OW970506.1) | 100% |
|  |  | *E. cloacae* isolate 114 (OW969715.1) | 100% |
|  |  | *E. cloacae* isolate 662 (OW969620.1) | 100% |
|  |  | *E. cloacae* isolate 114 (OW968296.1) | 100% |
